# Medication non-adherence as a driver of pharmaceutical waste: integrating top-down policies with bottom-up practice

**DOI:** 10.3389/fpubh.2025.1714049

**Published:** 2025-11-19

**Authors:** Przemyslaw Kardas, Tamas Agh

**Affiliations:** 1 Medication Adherence Research Center, Department of Family Medicine, Medical University of Lodz, Lodz, Poland; 2 Medication Adherence Research Group, Center for Health Technology Assessment and Pharmacoeconomic Research, University of Pécs, Pécs, Hungary

**Keywords:** medication adherence, drug wastage, environmental impact, health policy, sustainable healthcare, deprescribing, medication optimization, pharmaceutical pollution

## Abstract

Medication non-adherence is a widespread challenge affecting up to half of patients with chronic conditions, with profound implications for health outcomes, healthcare costs, and, increasingly recognized, environmental sustainability. Unused and improperly disposed medications contribute to pharmaceutical waste, overproduction, and pollution, amplifying the healthcare sector’s carbon footprint. This viewpoint highlights the need for coordinated action across clinical practice and health policy to mitigate this underappreciated dimension of environmental harm. We argue that addressing non-adherence is not solely a clinical imperative but also an ecological one, requiring dual responsibility: bottom-up engagement by healthcare professionals and patients, and top-down strategies embedded in policy and system-level reforms. Drawing on evidence from adherence interventions and sustainable prescribing initiatives, we outline actionable steps—from individualized medication optimization and deprescribing to public health campaigns and regulatory frameworks—to align adherence management with environmental goals. Tackling this problem offers a unique opportunity to improve patient outcomes while advancing climate-conscious healthcare and reducing overall healthcare-related costs. We call on clinicians, health systems, and policymakers to integrate adherence promotion into sustainability agendas and to view every prescription as both a therapeutic and environmental decision. Likewise, we urge optimization of environmentally safe and effective disposal systems for unused and expired drugs, ensuring that such measures become an integral part of comprehensive strategies to protect both human health and the planet.

## Introduction

Medication non-adherence, defined as the failure to take medications as prescribed (1), remains a widespread challenge, affecting approximately 50% of patients on chronic therapy (2). This burden is expected to rise further due to global population aging, increasing multimorbidity, and the expansion of defensive medical practices (3).

Medication non-adherence poses a major barrier to both patient health and the sustainability of healthcare systems. Over five decades of observational and interventional studies have consistently demonstrated its profound clinical and economic consequences (4). Suboptimal adherence is associated with adverse health outcomes, disease progression, and increased healthcare utilization, including hospitalizations—particularly among older adults and individuals with chronic conditions. At the individual level, non-adherence contributes to higher morbidity and poorer treatment outcomes (5). At the societal level, it leads to inefficient resource use, economic strain, and avoidable healthcare expenditures (6–8). The Organization for Economic Co-operation and Development (OECD) estimates that medication non-adherence has been associated with 200,000 deaths and EUR 125 billion avoidable medical expenditures per year in Europe (9). As a result, non-adherence represents not only a major public health concern but also a key barrier to realizing the full benefits of evidence-based therapies (3).

While these clinical and economic impacts have long been recognized, an equally profound yet largely overlooked consequence of medication non-adherence is its environmental impact (10, 11). In this paper, we discuss the evidence linking non-adherence to environmental harm and propose strategies to address this challenge, both at the level of individual clinical practice and within broader health policy.

## Ecological consequences of medication non-adherence

Pharmaceutical manufacturing, packaging, and distribution are highly carbon-intensive processes, substantially contributing to the environmental burden of healthcare (12). Pharmaceuticals alone contribute roughly 12% of the healthcare sector’s carbon footprint, which in turn accounts for about 5% of total emissions in major economies (13). Although evidence-based pharmacotherapy remains essential and its use is justified when it improves patient outcomes (14), this balance is disrupted when medications are prescribed but not used as intended.

**Figure 1:**
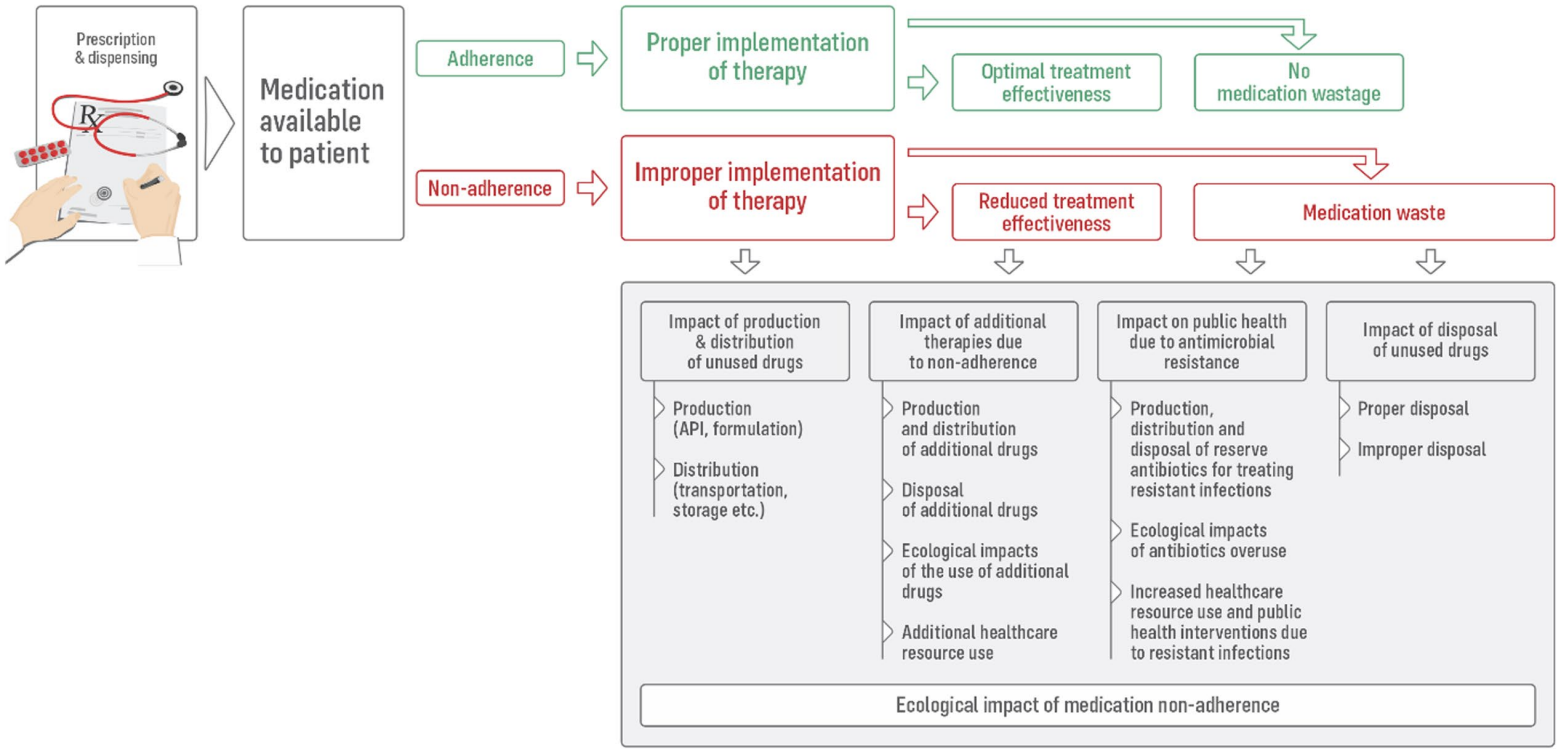
Ecological impact of medication non-adherence.

Beyond the well-established clinical and economic repercussions, medication non-adherence also initiates a cascade of environmental consequences (Figure 1). These include both the direct ecological impacts of unused medications and the broader effects arising from inefficiencies in treatment pathways. Importantly, these consequences emerge from deviations occurring across all three key phases of the medication-taking process (1): (i) Initiation—when therapy is commenced following the dispensing of the first prescription; (ii) Implementation—the daily execution of the prescribed treatment plan; and (iii) Continuation—the persistence of therapy over time, which in chronic conditions is typically intended to be indefinite.

Patients who delay or fail to initiate therapy, inconsistently follow the prescribed regimen (typically taking fewer doses than dispensed), or discontinue treatment prematurely face increased risk of poor health outcomes (4). This frequently necessitates additional diagnostic and therapeutic interventions, including hospitalizations and the introduction of supplementary medications. Such inefficiencies not only escalate healthcare costs but also intensify environmental burdens through the production, packaging, and distribution of extra pharmaceuticals, increased energy and other resources use within healthcare facilities, and additional medical waste generation.

The scale of the issue becomes particularly clear when viewed through the lens of pharmaceutical waste. Medication non-adherence is a key contributor (7, 14–18), significantly fueling the accumulation of unused medicines in households (18, 19). It is estimated to account for up to 50% of all discarded medications (16). A survey conducted in six European capital cities confirmed that the failure to follow prescribed treatments remains the most common reason for unused medicines (17). Supporting this, a detailed study of drugs returned to selected Italian pharmacies revealed that, on average, 68% of the original dosage units were still present in the packaging (19).

The proportion of household medications that are ultimately discarded may reach up to 50% (18), with significant consequences. The financial burden of this wastage is considerable: globally, unused medicines are estimated to account for 10–45% of household pharmaceutical expenditures (7). National data underscore the scale of the problem—for example, in Sweden, drug acquisition costs associated with non-adherence amount to SEK 11.2 billion (approximately EUR 1.2 billion) (8), while in the United Kingdom, annual losses from unused National Health Service (NHS) prescribed medicines are estimated at approximately £100–800 million (10).

However, the most profound repercussions of medication non-adherence may stem from its ecological impact, particularly through the disposal of unused or expired drugs. Importantly, even properly managed disposal carries an environmental burden. In those European countries in which well-established pharmaceutical take-back systems exist, unused household medications are typically returned to community pharmacies. There, they are deposited into secure collection containers and subsequently transported to centralized facilities for destruction via high-temperature incineration (20). While this approach prevents direct contamination of soil and water, it is resource-, and labor-intensive and generates significant carbon emissions through both transport and incineration, or even toxic pollution, thereby contributing to the healthcare sector’s carbon footprint (12). In France, the European leader in the collection of unused and expired medicinal products, the annual collection rate in 2018 was 259 g per capita, amounting to approximately 17,600 tonnes per year (11).

The challenge is compounded by the persistent prevalence of improper disposal practices, leading to pollution and toxicity. The most common method worldwide is the disposal of unused medications with household waste or by flushing them into sewage systems (21). This occurs even in high-income countries (7, 18, 20, 22)—for example, 68% of respondents in a Polish survey reported such practices (23), as did 83% in China (15). Similar patterns are observed in countries with established systems or programs for the return of unused drugs, suggesting that these measures are not sufficiently effective (20). The situation may be even more severe in low- and middle-income countries, in which take-back programs are infrequent (24).

Unfortunately, sewage treatment plants are generally not equipped to remove pharmaceutical contaminants from wastewater (20). As a result, these disposal habits contribute to the widespread contamination of terrestrial and aquatic ecosystems. Consequently, pharmaceutical residues—including antibiotics, analgesics, hormones, and non-steroidal anti-inflammatory drugs—are now detectable in surface waters, sediments, and soils across diverse geographical regions, with documented cases from, e.g., Canada, China, France, Sweden, and the United States (11, 15).

Such environmental contamination carries serious ecological and public health implications. Of particular concern is the improper disposal of antibiotics, which contributes significantly to the development and spread of antimicrobial resistance (AMR)—a major global health threat responsible for an estimated 33,000 deaths annually in Europe (11, 14, 15, 25). The United Nations Environment Program has identified pharmaceutical pollution from antibiotics as a critical driver of AMR (26). The presence of antibiotics, antifungals, and resistance genes in aquatic ecosystems accelerates the spread of resistant pathogens. Beyond microbial threats, adverse effects on wildlife have been observed, including fish mortality and endocrine disruption leading to intersex conditions in riverine species (11, 15, 27). These findings underscore the urgent need for integrated strategies that address both medication adherence and environmentally safe disposal pathways.

## Minimizing the ecological footprint of medication non-adherence

For the reasons discussed above, tackling medication non-adherence is not only a clinical and economic necessity but also a critical step toward reducing healthcare’s environmental footprint. Optimizing adherence may decrease the ecological impact associated with pharmaceutical production, distribution, and disposal (28).

Healthcare professionals are uniquely positioned to address this challenge by promoting responsible medication use and preventing avoidable waste. By implementing evidence-based adherence interventions, clinicians can simultaneously enhance patient outcomes, conserve healthcare resources, and mitigate the ecological harm associated with pharmaceutical waste. Fortunately, numerous studies have demonstrated the effectiveness of various strategies in enhancing adherence (29, 30).

For example, Medicines Optimization is a key strategy within the United Kingdom healthcare system designed to maximize the benefits of prescribed treatments. It builds upon the traditional concept of medicines management by emphasizing shared decision-making, regular medication reviews, and alignment of treatment with patients’ individual goals and circumstances. By integrating a patient-centered approach into routine practice, it ensures that medications are used effectively, safely, and sustainably to improve health outcomes (10, 11). Given that up to £300 million worth of medicines are unused and subsequently wasted each year in NHS primary and community care alone, tackling non-adherence in these settings could save approximately £150 million while also significantly reducing the environmental impact (27).

However, policymakers can also facilitate this process by shaping adequate supportive policies. In general, two complementary strategies are essential: reducing preventable medicine waste through improved adherence and ensuring safe disposal of unavoidable surplus medicines, thereby minimizing the release of pharmaceutical residues into the environment (15). In order to implement them effectively, both bottom-up and top-down approaches are necessary, as described in more detail in the following sections.

## The role of healthcare professionals in addressing medication non-adherence

**Figure 2:**
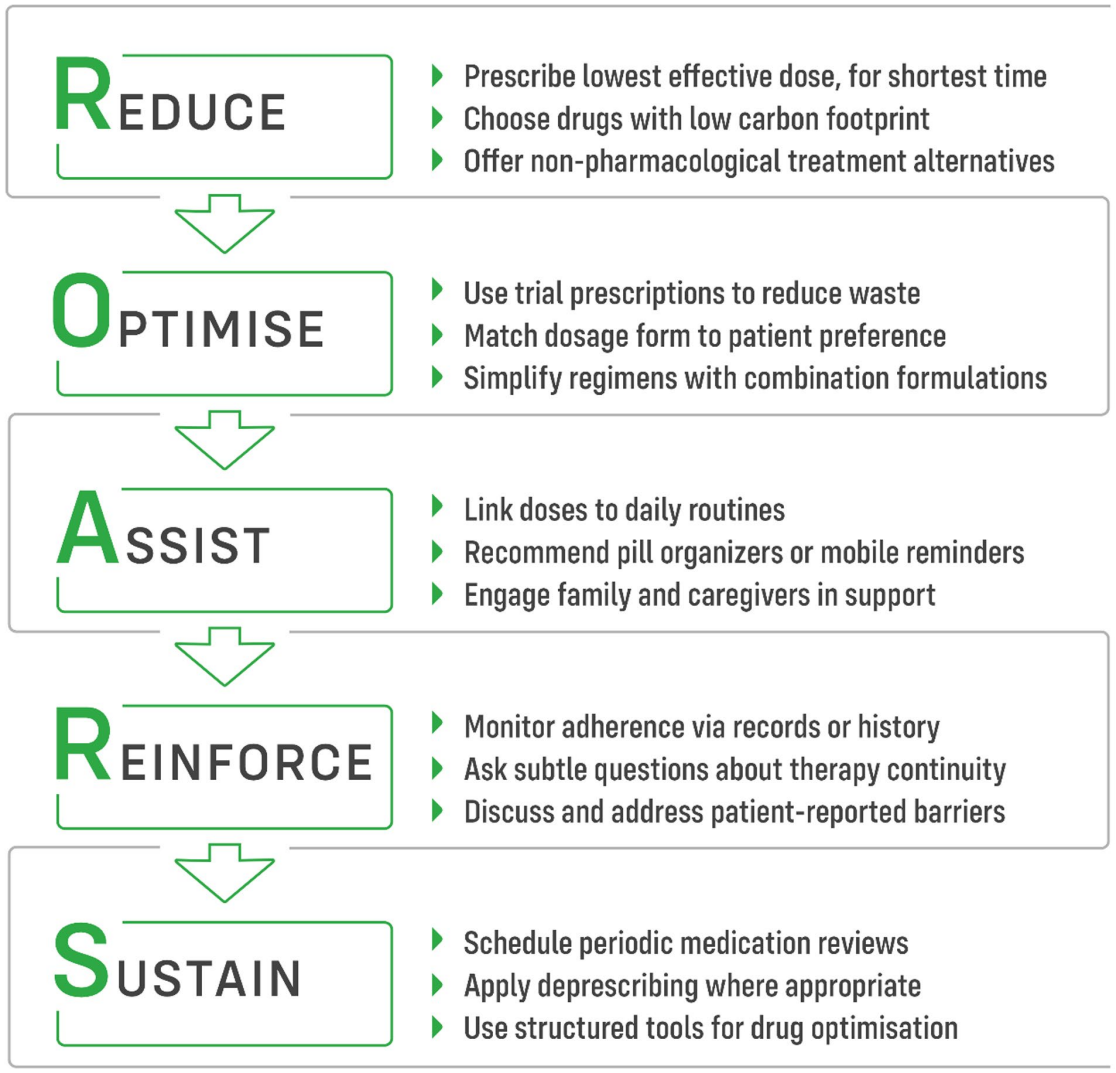
ROARS strategy of five practical steps for clinicians to reduce the ecological impact of medication non-adherence.

The actions that clinicians can take to reduce negative environmental effect of non-adherence could be described by 5-item ROARS strategy (Figure 2): Reduce–Optimize–Assist–Reinforce–Sustain, as outlined below.

### Reduce

Reducing the number of prescribed medications simplifies treatment regimens, enhances adherence, and mitigates the environmental impact of pharmaceutical production. Low-carbon prescribing involves offering the right people the right information so that they can choose the best treatment. It also means using the lowest effective dose for the shortest possible period of time, selecting drugs with the smallest carbon and ecological footprint, and making the best use of alternatives such as psychotherapy, etc. (11).

This approach can be effectively implemented through patient-centered care and shared decision-making. When co-developing personalized treatment plans with patients, HCPs should recognize that not every condition requires pharmacological intervention, even when a specific drug is available. Overcoming the pro-prescribing tendencies embedded in clinical guidelines necessitates well-informed patients, highlighting the crucial role of health literacy. Given the time constraints many prescribers face, interdisciplinary collaboration with trained healthcare professionals—such as nurses, pharmacists, and health educators—can help address this challenge, depending on local availability (31). Additionally, reliable online resources may serve as valuable tools for patient education (32).

When fully informed about the benefits and risks of a given medication, patients may, in some cases, choose to forgo pharmacological treatment (33)—for instance, opting out of preventive therapy for infrequent migraine attacks (34). Similarly, non-pharmacological approaches—including social prescribing (referral to community or statutory services), green prescribing (nature-based activities), and blue prescribing (water-based activities)—may offer efficacy comparable to that of, for example, antidepressants in managing mild mood disorders (11). Finally, providing patients with clear information on the clinical and ecological benefits of proper drug taking can also improve outcomes and adherence (33).

### Optimise

Building on a mutually agreed list of medications, healthcare professionals can further optimize therapy to enhance adherence and support sustainability. In the United Kingdom and several other countries, these efforts are integrated into the New Medicine Service (NMS) or similar programs (35). The NMS provides structured follow-up and counseling by community pharmacists for patients starting new treatments for chronic conditions, helping to identify early adherence barriers, manage side effects, and reinforce patients’ understanding of their therapy.

In cases where adverse effects are anticipated, a useful recommendation is to opt for ‘trial prescriptions’—small initial quantities that enable prescribers to evaluate the medication’s suitability for the patient before committing to a full course, reducing the risk of waste, and significantly reducing the costs incurred, e.g., due to oncological therapy (33, 36). By considering patient preferences, healthcare professionals can select dosage forms that are more acceptable by individual patient, such as capsules instead of tablets, or suppositories instead of injections, ensuring better treatment acceptance (37). Further simplification, such as the use of multi-compound tablets for cardiovascular conditions or combination inhalers for respiratory diseases, not only improves treatment adherence but also provides environmental benefits by reducing packaging and transportation-related emissions (38). Additionally, choosing dry powder inhalers (DPIs) over metered-dose inhalers (MDIs) can support adherence, as DPIs do not require coordination between drug release and inhalation, thereby promoting more effective inhalation technique. At the same time, they significantly reduce the release of powerful greenhouse gases that contribute significantly to climate change, as many MDIs still rely on hydrofluorocarbon propellants (33, 39).

### Assist

Both prescribers, as well as the other healthcare professionals, may play an active role in ensuring that patients successfully implement their prescribed therapy. By promoting self-monitoring and helping patients establish cues for medication intake—such as linking it to daily routines like meals or tooth brushing—they can significantly reduce unintentional non-adherence due to forgetfulness (40). Recommending the use of technical aids, such as pill organizers and automated dispensers, not only helps prevent missed doses but also facilitates feedback mechanisms, such as SMS reminders or phone calls (3). These systems can also notify caregivers or family members, even remotely, ensuring additional support. Similarly, various mobile applications—many of which are freely available—offer comparable benefits, with even lower carbon footprint (41). Actively engaging family members and caregivers in the patient’s treatment journey is highly valuable, as social support has been shown to enhance adherence (42).

### Reinforce

Medication adherence tends to decline over time, often mirroring a decrease in patients’ motivation to maintain long-term therapies. This is particularly evident in low- and asymptomatic conditions such as hypertension, type 2 diabetes, and hyperlipidemia (43). Healthcare professionals can take a proactive approach in identifying signs of therapy discontinuation by reviewing sources such as electronic patient records and prescribing or dispensing histories (44, 45). Subtle, non-intrusive questioning during consultations may also help uncover lapses in adherence. When such cases arise, the key consideration is understanding the patient’s reason for non-persistence and exploring, through open discussion, whether this barrier can be addressed.

### Sustain

Healthcare professionals providing long-term care, such as family physicians and community pharmacists, may also help adherence in long-term perspective, adopting iterative approach. As patients’ priorities shift, motivation fluctuates, and new barriers emerge, continuous reassessment becomes essential. Therefore, employing periodically strategies such as medication reviews and deprescribing is particularly valuable (33, 46, 47). For patients with multimorbidity, a structured approach to drug optimization is recommended, utilizing tools such as the Beers Criteria, STOPP-START, or the Scottish 7-Step Approach—many of which are available in digital formats to aid clinical decision-making (47).

## The role of policy-level strategies in addressing medication non-adherence

Policymakers, governing bodies, payers, and other stakeholders play a crucial role in reducing medication non-adherence. By establishing supportive policies, funding adherence-enhancing programs, and coordinating public awareness efforts, they can provide the structural conditions needed for optimal medication use and reduced pharmaceutical waste. Currently, however, patients often depend largely on the goodwill of their healthcare providers to receive help in following their prescribed therapies.

According to a recent OECD report, medication adherence is far from being a priority on national health agendas. Most European countries neither systematically monitor adherence nor take regular actions to improve it (9). The scarcity of reimbursed medication adherence–enhancing interventions across Europe is striking (48). This is particularly frustrating, given that numerous adherence-focused interventions are well documented in the literature (29, 49–51).

When deciding which intervention to implement and fund, decision-makers should prioritize those supported by robust evidence, with particular attention to long-term impact. A recent purposive umbrella review identified eight types of effective patient-directed interventions that are readily implementable in the United Kingdom NHS: pharmacist-delivered support; face-to-face counseling; combination drug formulations; reminder and prompting systems; personalized adherence feedback; habit-promoting strategies; self-management and behavior-enhancement techniques; and multi-component interventions delivered in parallel (52).

Unfortunately, implementation of such interventions may face difficulties. Despite proven clinical value, incentives remain limited for developing and approving fixed-dose combination tablets, particularly for generic medications. In markets such as the United States, regulatory approval processes are often cumbersome even when the efficacy of individual component drugs is well established. Hence, streamlining such pathways and encouraging manufacturers through targeted incentives could expand access to adherence-supportive formulations globally.

An innovative approach, already adopted in the United States, involves rewarding healthcare providers when at least 80% of their patients achieve high adherence levels. Evidence shows that this model can significantly improve adherence rates (53). Building on this concept, pharmaceutical companies could be made co-responsible for adherence—e.g., by linking a certain minimum adherence level to eligibility for drug reimbursement. A parallel can be drawn with the successful implementation of Extended Producer Responsibility (EPR) schemes for the collection and disposal of unused or expired medicines, which operate effectively in several European countries (20).

## Discussion

In this paper, we summarize the major ecological consequences of medication non-adherence. Numerous profound impacts indicate that this problem is more than a clinical or economic concern—it is an environmental issue of urgent importance. Each unused pill, capsule, or inhaler represents not only a missed therapeutic opportunity but also the waste of manufacturing energy, packaging materials, transportation fuel, and, ultimately, resources for disposal. When aggregated across millions of prescriptions, the cumulative effect on the healthcare sector’s carbon footprint is substantial. This environmental dimension has remained largely invisible in the medication non-adherence debate, yet, given the high prevalence of non-adherence, it is inseparable from the broader goals of sustainable healthcare.

The primary and most effective environmental intervention is to prevent pharmaceutical waste at its source by improving adherence. Evidence-based strategies—ranging from medication optimisation and deprescribing to the use of reminder systems and patient education—can ensure that medicines dispensed are medicines used, thereby reducing unnecessary production and waste. In this sense, promoting adherence is not simply good clinical practice; it is also climate-conscious practice. The message is clear: every prescription should be viewed simultaneously as a therapeutic decision and an environmental responsibility.

This dual lens calls for a coordinated response. At the clinical level, healthcare professionals should be supported to integrate adherence-enhancing strategies into routine practice, using structured reviews, shared decision-making, and multidisciplinary approaches to tailor therapy to patients’ real-world capabilities and preferences. At the system level, policymakers must embed adherence into national sustainability and health performance frameworks, allocate funding for proven adherence-enhancing interventions, and monitor their long-term impact. Without policy alignment, efforts will remain fragmented and dependent on individual goodwill.

However, even in the most successful adherence scenarios, a certain volume of unused medicines is inevitable—due to changes in therapy, adverse effects, or the death of a patient. This surplus must be managed responsibly. Current evidence shows that improper disposal, such as throwing medicines into household waste or flushing them down the toilet, remains widespread even in high-income countries with established collection systems (20). These practices contaminate soil and water, disrupt ecosystems, and contribute to antimicrobial resistance, an urgent global health threat.

Proper disposal through pharmacy-based take-back schemes or municipal collection programs is essential, yet it should be understood as a secondary line of defense—necessary to mitigate harm from unavoidable waste, but not a substitute for reducing waste through improved adherence. Moreover, disposal systems themselves carry an environmental cost, as high-temperature incineration and transportation produce greenhouse gas emissions and, in some cases, toxic residues. Therefore, while proper disposal is vital, the overarching strategy must focus first on minimizing waste generation.

In conclusion, there is one more reason to tackle medication non-adherence: it offers a rare win–win–win scenario—better health outcomes, lower healthcare costs, and reduced environmental harm. By embedding adherence promotion into sustainability agendas and coupling it with effective disposal strategies, health systems can make every prescription count—for patients, public health, and the planet. The time for recognizing non-adherence as an ecological challenge is long past; the time for coordinated, decisive action is now.

## Data Availability

The original contributions presented in the study are included in the article/supplementary material, further inquiries can be directed to the corresponding author.
